# Reference interval for serum amyloid a in apparently healthy donkeys measured with a donkey-specific ELISA

**DOI:** 10.3389/fvets.2026.1884314

**Published:** 2026-07-13

**Authors:** Maciej Perzyna, Edith Bardet, Katarzyna Baranowska, Paula Kiełbik, Maria Puchalska, Bartosz Pawliński, Olga Witkowska-Piłaszewicz

**Affiliations:** 1 Department of Large Animals Diseases and Clinic, Institute of Veterinary Medicine, Warsaw University of Life Sciences, Warsaw, Poland; 2 Scientific Society of Veterinary Medicine Students, Warsaw University of Life Sciences, Warsaw, Poland; 3 Department of Pathology and Veterinary Diagnostic, Institute of Veterinary Medicine, Warsaw University of Life Sciences, Warsaw, Poland

**Keywords:** acute phase proteins, ASVCP/CLSI guidelines, biomarker of inflammation, donkeys, reference interval, species-specific values

## Abstract

**Introduction:**

Serum amyloid A (SAA) is a major acute phase protein used as an inflammatory biomarker in equine medicine. However, no universally accepted assay-specific reference interval (RI) exists for donkeys, and available literature remains limited, with only few studies using different donkey breeds and immunoassay platforms. This study aimed to establish an assay- and population-specific RI for serum SAA in apparently healthy donkeys using a species-specific double sandwich ELISA.

**Methods:**

Serum SAA concentrations were measured in 176 apparently healthy donkeys. RI estimation followed ASVCP/CLSI recommendations. The primary RI was calculated nonparametrically as the central 95% interval from all reference individuals, and two-sided 90% confidence intervals (CIs) for the reference limits were estimated nonparametrically. Data were inspected using Tukey’s interquartile fences; however, high-end observations were not excluded from the primary RI analysis. Exploratory analyses were performed to assess the need for partitioning by sex, age, or breed.

**Results:**

The nonparametric RI for serum SAA was 2.91–42.85 ng/mL. The 90% CI for the lower reference limit was 0.00–4.07 ng/mL, and the 90% CI for the upper reference limit was 35.49–67.10 ng/mL. Exploratory analyses did not support partitioning by sex, age, or breed. The relatively broad CI for the upper reference limit indicated imprecision, likely reflecting the influence of a minority of high-end observations.

**Discussion:**

The low SAA concentrations observed in this study are consistent with findings reported in horses. Differences between this and previous donkey studies may reflect variation in assay platform, sample size, management conditions, physiological state, or population characteristics. Because SAA is a rapidly responsive acute phase protein, the proposed interval should be interpreted as an assay- and population-specific RI rather than as a disease-classification decision limit. Clinical interpretation should therefore always consider the individual donkey’s clinical context.

## 1 Introduction

Donkeys (*Equus asinus*) are increasingly recognized as a distinct equid species with specific physiologic and clinical characteristics (1). Their population continues to increase, especially in developing world regions, such as Africa. This is related to the fact that donkeys play a crucial role as working animals, supporting the daily life of millions of people and being an economically and ecologically important livestock species (2–4). Working equids are predisposed to several workload-associated conditions, most notably lameness, colic, back pain, poor body condition, and parasitic infestations (5, 6). In addition, donkeys may serve as food-producing animals, providing milk and meat. Beyond their work and food-related functions, donkeys may also be kept as companion animals or used in animal-assisted therapy (onotherapy), thereby supporting human mental and physical health (7, 8). Therefore, the development of scientific research focused on improving the welfare and veterinary care of these animals is essential (9). Although donkeys share many disorders with horses, diagnostic assessment in donkeys may be challenging because clinical signs can be subtle and nonspecific, and affected animals may mask pain or systemic illness until disease is advanced (1, 10). In this context, objective biomarkers that reflect inflammation may support earlier recognition of disease, improve triage decisions, and facilitate monitoring of response to therapy (11, 12). Tests appropriate for monitoring the onset of inflammatory responses should ideally be rapid and straightforward to perform, enabling timely support for donkey breeders and veterinarians. Measuring blood levels of proteins expressed during the acute phase response of inflammation (acute phase proteins) meets many of these requirements (13, 14).

Acute phase proteins (APPs) are nonspecific inflammatory biomarkers produced primarily by the liver in response to pro-inflammatory cytokines (15). Among equine APPs, serum amyloid A (SAA) is considered a major APP due to its rapid kinetics, large magnitude of increase, and relatively short half-life, making it suitable for detecting and monitoring inflammatory processes (16). In healthy horses, baseline SAA concentrations are typically very low, while concentrations can increase markedly in response to infection, tissue injury, or systemic inflammation (15). Additionally, SAA levels may be altered in horses during the course of gastrointestinal disorders, such as colic (17, 18) or musculoskeletal conditions, including septic arthritis (19, 20). Comparable use of SAA in donkey medicine is promising; however, only a limited number of studies are available, and standardized reference values have not been established (10, 11, 14, 21, 22). The interpretation of SAA cannot rely on reference values established for horses and must be considered within a donkey-specific context, because APP magnitude, baseline concentrations, and analytical performance can differ across species and assays (11, 12, 14, 21, 22). In equids, fibrinogen is also widely used as an acute phase marker, particularly in the assessment of inflammatory and infectious conditions, although its kinetics are generally slower than those of SAA (15). Other APPs, including haptoglobin and albumin, may also provide clinically relevant information, with albumin acting as a negative APPs (15–17). Nevertheless, SAA is considered especially useful for early and dynamic monitoring of inflammatory responses because of its rapid increase and short half-life (15, 16).

Available studies indicate that SAA concentrations in donkeys increase primarily during endotoxemia (11, 21), particularly in the early phase of the condition, and decrease following anti-inflammatory treatment, supporting its usefulness for monitoring therapeutic response (11). Changes in SAA have also been reported during the periparturient period in jennies and foals (22), in working donkeys (10), and in recently captured feral donkeys (14), where inflammation and stress are relevant factors (23). In contrast, no significant changes in SAA were observed after acute bleeding, likely due to a short sampling period (24). Importantly, several studies were conducted in small cohorts (e.g., *n* = 8), which limits the strength of the conclusions (24).

Current evidence remains limited for the general donkey population, and results obtained with different analytical platforms are not directly interchangeable. Assay-specific RIs derived from well-characterized, apparently healthy donkeys are needed. The aim of this study was to establish an assay-specific RI for serum SAA in healthy, pasture-managed donkeys using a donkey-specific ELISA and to explore whether partitioning by sex, age, or breed was supported in this cohort.

## 2 Materials and methods

### 2.1 Animals

The study included 176 apparently healthy donkeys (*Equus asinus*): 92 jennies, 46 geldings, and 38 jacks, aged 0.3–35 years (median 5.0 years; IQR 3.0–8.3 years). The animals were maintained under comparable pasture-based management conditions. Apparent health status was established by veterinary physical examination performed at the time of sampling, together with a review of recent history. Routine hematological and biochemical profiles were performed, and they were within reference values. All 176 animals fulfilled the predefined *a priori* inclusion criteria and were retained as the reference cohort for the primary RI analysis. For exploratory subgroup analyses, age was considered both as a continuous variable and in predefined age classes: <1 year (*n* = 6), 1–3 years (*n* = 49), 4–10 years (*n* = 85), 11–20 years (*n* = 31), and >20 years (*n* = 5). Breed categories comprised standard-sized donkeys (*n* = 138), miniature donkeys (*n* = 25), and Poitou donkeys (*n* = 13). To further explore age-related trends, an additional age-based classification was applied, comprising young donkeys (<4 years; *n* = 56), adult donkeys (4–20 years; *n* = 115), and geriatric donkeys (>20 years; *n* = 5).

### 2.2 Selection of reference individuals

The determination of the population-based reference interval (RI) followed the recommendations of the American Society for Veterinary Clinical Pathology (ASVCP) and the Clinical and Laboratory Standards Institute (CLSI) (25). Reference individuals were selected using a priori inclusion and exclusion criteria based on clinical status, recent medical history, sample quality, and management conditions (Table 1). The reference population was intended to represent apparently healthy donkeys managed under comparable pasture-based conditions.

**Table 1:** *A priori* inclusion and exclusion criteria used to define the reference population for SAA RI determination.

Examples of inclusion criteria	Examples of exclusion criteria
Apparently healthy donkeys verified by physical examination, recent history, and routine hematological and biochemical screening. Juvenile and adult animals representing the target population (0.3–35 years). Both sexes and the represented breeds included. Animals raised under similar environmental and management conditions (e.g., pasture-based systems). No recent history of illness or medical treatment within the last 3 months.	Abnormal findings during clinical examination (e.g., fever, lameness, respiratory or gastrointestinal abnormalities). Current medication or recent therapeutic intervention. Marked preanalytical/sample-quality problems (e.g., hemolysis, lipemia, inadequate sample quality). Known systemic disease or physiologic state outside the target reference population.

### 2.3 Sampling

All of the procedures of blood sampling were performed as part of routine health examinations, and thus, according to the European directive EU/2010/63 and Polish regulations regarding experiments on animals, there was no need for the approval of the Ethics Committee for the described procedures, which qualified as non-experimental clinical veterinary practices and were excluded from the directive. Blood samples were collected from the jugular vein of each donkey using BD Vacutainer (Becton Dickinson, UK) system with dry (serum) tubes to ensure adequate sample volume and minimize discomfort. The blood collection was conducted during one season (summer) in the morning to control for potential variations due to seasonal and diurnal factors. Serum samples were allowed to clot at room temperature for 1 h, centrifuged at 3000 × g for 15 min, and stored at −80 °C until further analyses.

### 2.4 Laboratory analysis

Serum SAA concentrations were measured using a donkey-specific double-antibody sandwich ELISA (Donkey Serum Amyloid A [SAA] ELISA Kit; Enlibio Biotech Co., Ltd., Wuhan, China) according to the manufacturer’s instructions. The stated analytical sensitivity was 0.5 ng/mL, and the working range was 1.56–100 ng/mL. Intra assay Precision is ≤ 8% and Inter assay Precision ≤ 12%. Intra-assay precision was additionally assessed in-house using 4 replicate measurements of low-, medium-, and high-concentration samples (Supplementary Table S1). The obtained coefficients of variation indicated acceptable within-run repeatability. Results are reported throughout in ng/mL. The absorbance was measured by a Multiscan Reader (Labsystem, Helsinki, Finland) using a Genesis V3.00 software program.

### 2.5 Data analysis

Statistical analyses were performed on the full reference cohort (*n* = 176). Serum amyloid A concentrations were summarized as mean ± SD and median (Q1-Q3). Distributional shape was assessed by histogram, normal Q-Q plot, and the Shapiro–Wilk test; however, normality assessment was descriptive only and did not determine the primary RI method. In accordance with ASVCP/CLSI recommendations for datasets containing at least 120 reference individuals, the primary RI was prespecified as a nonparametric central 95% interval. The lower and upper reference limits were estimated as the 2.5th and 97.5th fractiles from the ordered observations, and 2-sided 90% confidence intervals (CIs) for the reference limits were derived from binomial order statistics. Potential outliers were explored using Tukey’s interquartile fences. Two observations of 0.00 ng/mL and 17 observations above the upper fence were reviewed, but no observation was excluded from the primary RI analysis because no analytical/preanalytical error or independent clinical reason for exclusion was documented. A sensitivity analysis was then performed after excluding the 17 upper-tail observations to quantify their influence on the RI. Exploratory subgroup analyses were conducted only to assess whether RI partitioning might be warranted and were not used to derive subgroup-specific RIs. Because SAA values were non-Gaussian and subgroup sizes were unequal, differences among sex, breed, and age-class groups were assessed using Kruskal–Wallis tests, and the association between SAA and age as a continuous variable was assessed using Spearman’s rank correlation. Here, W denotes the Shapiro–Wilk test statistic, H denotes the Kruskal–Wallis rank-sum test statistic, and rho denotes Spearman’s rank correlation coefficient. Statistical significance was set at *p* < 0.05 (two-sided).

Also, the summary of the values of SAA from all available peer-reviewed scientific papers on this topic (10–12, 14, 21, 22, 24, 26), as well as this study’s mean values, has been performed (Table 2).

**Table 2:** Summary of baseline SAA concentration values from all available peer-reviewed scientific papers.

SAA concentration references	Number of studied animals (*n*)	Type of studied population
7.8 ± 2.1 μg/mL (11)	6	Male miniature donkeys
10.80 mg/L (14)	85	Feral jennies and jacks removed from the Death Valley
0.1–0.6 mg/L (21)	73	Andalusian donkeys
25.95 ± 2.39 ug/mL (22)	10	Jennies within 48 h from delivery
14.99 ± 1.79 ug/mL (22)	10	Jennies at 30 days of lactation
37.44 ± 19.76 ug/mL (22)	10	Male and female newborn donkey foals (within 48 h from birth)
16.04 ± 18.14 ug/mL (22)	20	Male and female donkey foals (within 1 month of age)
318 ± 77 ng/mL (24)	8	Male and female adult donkeys aged 8.5 ± 1.6 years

## 3 Results

### 3.1 SAA RI in apparently healthy donkeys

**Figure 1:**
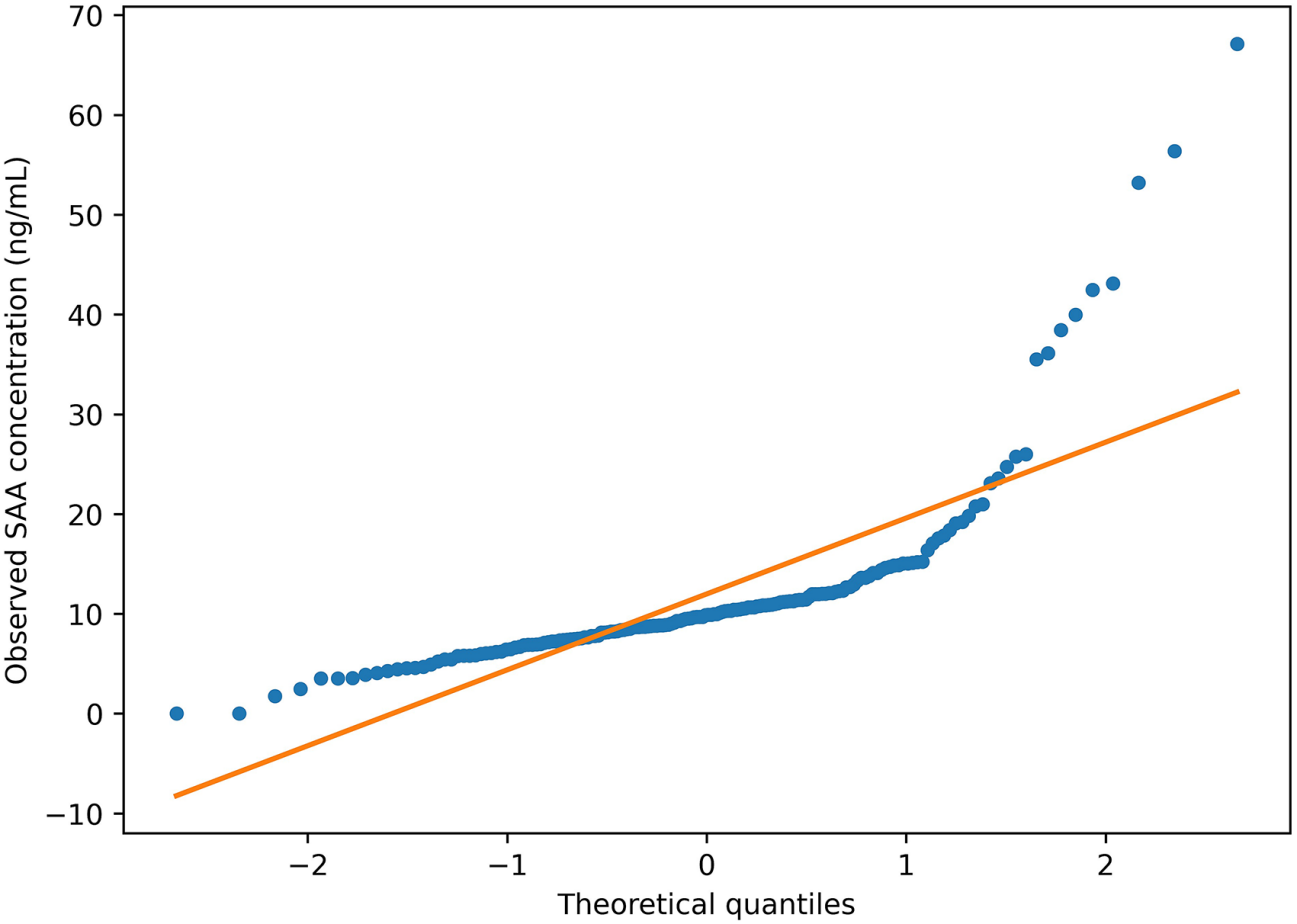
Histogram of serum amyloid A (SAA) concentrations in the full reference cohort of apparently healthy donkeys (*Equus asinus*) (*n* = 176). Dashed vertical lines indicate the lower and upper nonparametric reference limits (2.91 and 42.85 ng/mL, respectively).

**Figure 2:**
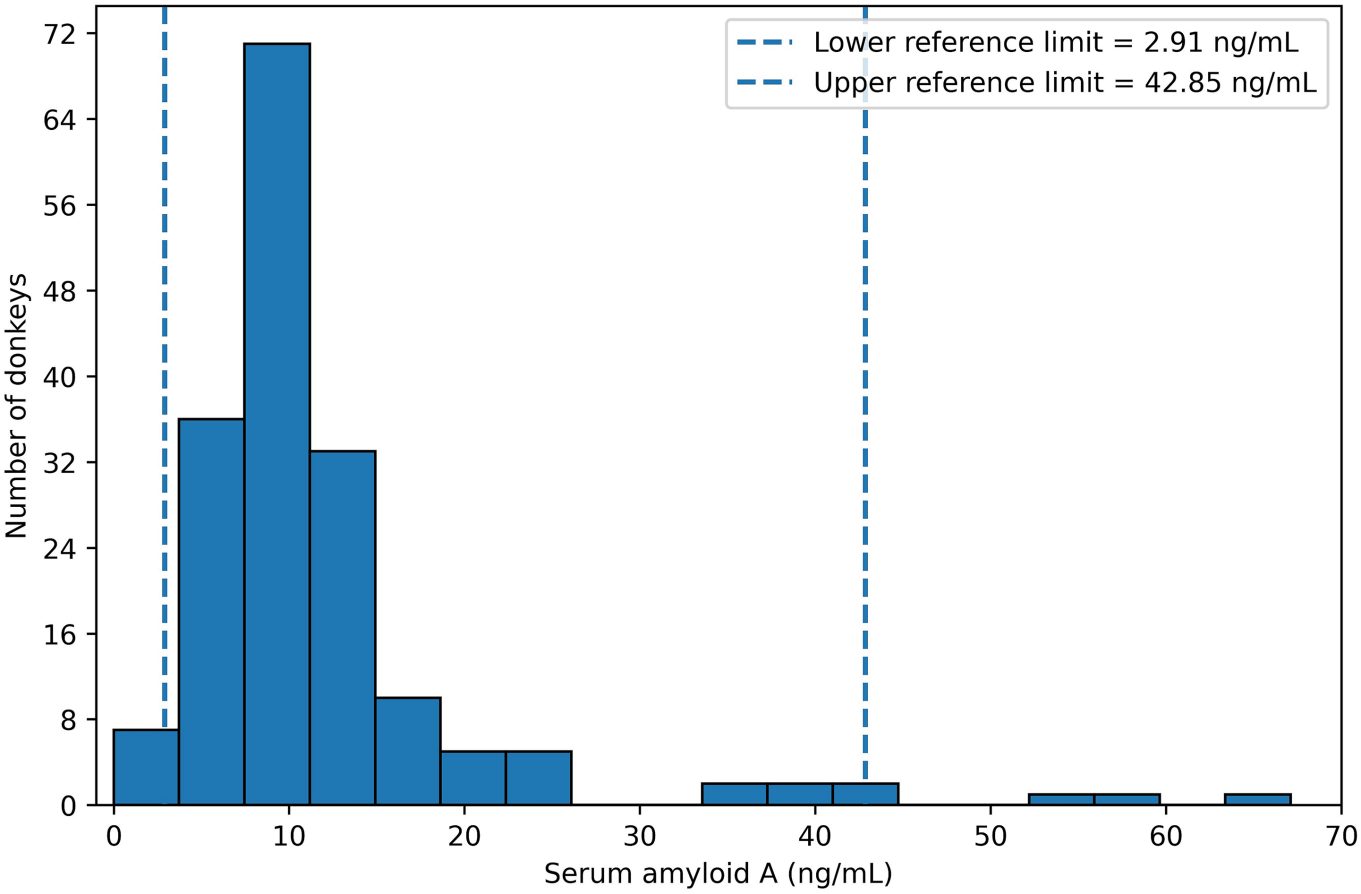
Normal Q–Q plot of serum amyloid A (SAA) concentrations in apparently healthy donkeys (*Equus asinus*) (*n* = 176). Deviation from the reference line, particularly in the upper tail, indicates a non-Gaussian distribution.

Serum SAA concentrations were measured in 176 apparently healthy donkeys. The distribution was right-skewed, with observed values ranging from 0.00 to 67.10 ng/mL (median 9.88 ng/mL; IQR 7.47–12.29 ng/mL; mean ± SD 11.99 ± 9.32 ng/mL). Histogram and Q–Q plot showed clear deviation from normality, which was confirmed by the Shapiro–Wilk test (*W* = 0.660, *p* < 0.001) (Figures 1, 2). Seventeen observations exceeded the upper Tukey IQR threshold, and two observations were 0.00 ng/mL; all were retained in the primary RI analysis. Using the full reference cohort and the prespecified ASVCP-compliant nonparametric approach, the RI for serum SAA was 2.91–42.85 ng/mL (Table 3). The 90% CI for the lower reference limit was 0.00–4.07 ng/mL, and the 90% CI for the upper reference limit was 35.49–67.10 ng/mL (Figure 1, Table 3). In the sensitivity analysis excluding the 17 upper-tail observations, the RI narrowed to 2.46–17.83 ng/mL, with 90% CIs of 0.00–3.90 ng/mL for the lower reference limit and 15.22–19.21 ng/mL for the upper reference limit.

**Table 3:** ASVCP/CLSI-compliant reference interval summary for serum amyloid A (SAA) in apparently healthy donkeys (*Equus asinus*).

Item	Value
Reference population	Apparently healthy pasture-managed donkeys (*Equus asinus*)
Sample size	176
Analyte and matrix	Serum amyloid A in serum
Assay/method	Donkey-specific double-antibody sandwich ELISA; Enlibio Biotech Co., Ltd.; 96-well format
Interval definition	Central 95% RI; 2.5th and 97.5th percentile limits
Primary statistical method	Nonparametric; two-sided 90% CIs from binomial order statistics
Observed range	0.00–67.10 ng/mL
Distribution	Right-skewed; Shapiro–Wilk *W* = 0.660, p < 0.001
Lower reference limit	2.91 ng/mL (2.5th percentile; 90% CI: 0.00–4.07 ng/mL)
Upper reference limit	42.85 ng/mL (97.5th percentile; 90% CI: 35.49–67.10 ng/mL)
Primary RI	2.91–42.85 ng/mL
High-end observation assessment	17 observations exceeded the upper Tukey IQR threshold; all were retained because no documented analytical, preanalytical, or clinical reason for exclusion was identified
Sensitivity analysis	After excluding the 17 upper-tail observations: RI 2.46–17.83 ng/mL; lower 90% CI 0.00–3.90 ng/mL; upper 90% CI 15.22–19.21 ng/mL

### 3.2 Exploratory subgroup analyses by sex, age category and breed

**Figure 3:**
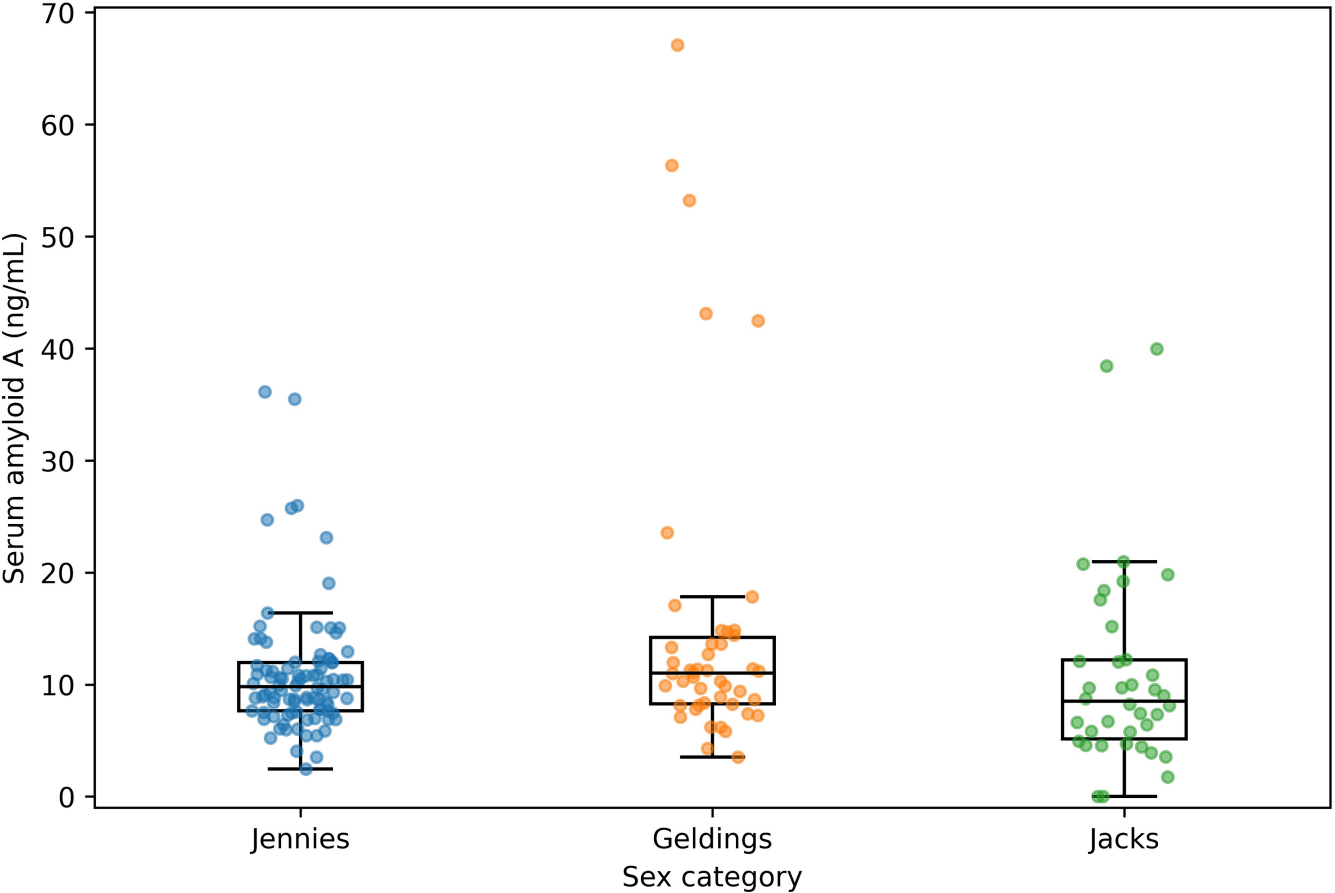
Boxplots of serum amyloid A (SAA) concentrations according to sex category in the full reference cohort of apparently healthy donkeys (*Equus asinus*) (*n* = 176). Jittered points represent individual animals.

Exploratory subgroup analyses were performed in the full dataset (Table 4). SAA did not differ significantly among sex categories (Kruskal–Wallis *H* = 5.79, *p* = 0.055). Median (Q1-Q3) and mean ± SD SAA concentrations were 9.81 (7.65–11.97) and 10.89 ± 5.66 ng/mL in jennies (*n* = 92), 11.03 (8.27–14.21) and 15.21 ± 13.98 ng/mL in geldings (*n* = 46), and 8.50 (5.14–12.19) and 10.77 ± 8.78 ng/mL in jacks (*n* = 38) (Figure 3, Table 4). No association between age and SAA was detected (Spearman’s rho = −0.040, *p* = 0.599), and SAA did not differ significantly across predefined age classes (<1 year, *n* = 6; 1–3 years, *n* = 49; 4–10 years, *n* = 85; 11–20 years, *n* = 31; >20 years, *n* = 5) (Kruskal–Wallis *H* = 1.91, *p* = 0.752). Mean ± SD SAA concentrations by age class were 10.76 ± 6.51 ng/mL in young donkeys (<4 years; *n* = 56), 12.72 ± 10.52 ng/mL in adult donkeys (4–20 years; *n* = 115), and 8.83 ± 3.84 ng/mL in geriatric donkeys (>20 years; *n* = 5). SAA also did not differ significantly among breed categories (Kruskal–Wallis *H* = 2.80, *p* = 0.246). Median (Q1–Q3) and mean ± SD SAA concentrations were 9.69 (7.49–12.02) and 11.79 ± 9.30 ng/mL in standard-sized donkeys (*n* = 138), 10.92 (7.83–15.22) and 13.84 ± 10.01 ng/mL in miniature donkeys (*n* = 25), and 9.30 (7.46–10.51) and 10.50 ± 8.35 ng/mL in Poitou donkeys (*n* = 13). Overall, these exploratory analyses did not support partitioning of the RI by sex, age, or breed in this cohort.

**Table 4:** Exploratory subgroup summary of serum amyloid A (SAA) concentrations in apparently healthy donkeys (*Equus asinus*).

Factor	Group	*n*	Mean ± SD (ng/mL)	Median (Q1-Q3) (ng/mL)	Range (ng/mL)	Test statistic	*p*-value
Sex	Jennies	92	10.89 ± 5.66	9.81 (7.65–11.97)	2.46–36.13	Kruskal–Wallis *H* = 5.79	0.055
Sex	Geldings	46	15.21 ± 13.98	11.03 (8.27–14.21)	3.53–67.10		
Sex	Jacks	38	10.77 ± 8.78	8.50 (5.14–12.19)	0.00–39.98		
Age	Young (<4 years)	56	10.76 ± 6.51	9.89 (7.22–12.08)	0.00–42.47	Kruskal–Wallis *H* = 1.26	0.532
Age	Adult (4–20 years)	115	12.72 ± 10.52	9.96 (7.72–12.50)	1.76–67.10		
Age	Geriatric (>20 years)	5	8.83 ± 3.84	8.48 (7.56–8.49)	4.55–15.07		
Breed	Standard-sized	138	11.79 ± 9.30	9.69 (7.49–12.02)	0.00–67.10	Kruskal–Wallis *H* = 2.80	0.246
Breed	Miniature	25	13.84 ± 10.01	10.92 (7.83–15.22)	4.07–53.21		
Breed	Poitou	13	10.50 ± 8.35	9.30 (7.46–10.51)	1.76–36.13		

## 4 Discussion

This study establishes a serum SAA RI for apparently healthy donkeys measured with a donkey-specific ELISA. As RI is influenced by the applied analytical method, the use of a species-specific double-sandwich ELISA, characterized by high analytical sensitivity and specificity, supports the quality of the obtained SAA measurements (25, 27). Using all 176 reference individuals and ASVCP-compliant analysis, the RI was 2.91–42.85 ng/mL. The wide upper 90% confidence interval indicates that the upper reference limit is imprecise and strongly influenced by a minority of high-end observations, which is consistent with the biological behavior of SAA as a rapidly responsive acute phase protein (15, 16).

Seventeen observations exceeded the upper Tukey fence. In accordance with the ASVCP-guided primary analysis plan, these observations were retained in the primary nonparametric RI analysis because no independent analytical, preanalytical, or clinical reason for exclusion was documented. However, sensitivity analysis excluding these observations yielded a substantially narrower RI (2.46–17.83 ng/mL), indicating that the upper reference limit is sensitive to high-end values in this dataset.

Based on these exploratory analyses, there was no evidence supporting partitioning of the SAA RI by age or breed in this dataset. These exploratory analyses did not identify a sufficiently strong signal to justify RI partitioning in this dataset; however, the subgroup sizes were limited for formal subgroup-specific RI derivation. A potential sex-related difference was observed for intact males (jacks) showing lower baseline SAA compared with jennies and geldings; however, subgroup sizes (notably jacks, *n* = 38) remain below typical minimum recommendations for establishing separate subgroup-specific reference intervals, and this finding should be interpreted cautiously and confirmed in larger cohorts. These data provide an assay-specific baseline for interpreting SAA concentrations in donkeys evaluated in similar management conditions and can support both clinical decision-making and study design (15, 25).

Comparison with previous donkey studies should remain cautious because reported SAA concentrations vary substantially with assay platform, calibration, biological matrix, physiologic state, and study design. This is especially relevant for SAA, for which values in healthy or mildly stressed animals may be very low in some studies and considerably higher in others. The present data therefore support the use of assay- and laboratory-specific intervals rather than application of a universal donkey threshold (10–12, 14, 21, 22, 24, 26). Since SAA is recognized in veterinary medicine as a useful diagnostic and treatment monitoring tool, this parameter has been measured in donkeys under various medical conditions. In one experimental study on miniature donkeys, baseline SAA concentration determined via sandwich ELISA was approximately 7.8 μg/mL in control animals, followed by a marked increase to 23.4 μg/mL within only 6 h of endotoxemia induction (11). An important aspect to consider is the potential influence of experimental protocols on baseline SAA concentrations. Even when animals are classified as clinically healthy, experimental procedures such as food and water restriction, relocation, handling, and restraint may act as physiologic stressors. Thus, they could transiently increase inflammatory biomarkers, including SAA. In a separate study, the median SAA concentration of around 0.35 mg/L was reported in healthy adult Andalusian donkeys using validated immunoassays. Following endotoxemia induction in a smaller cohort, SAA concentrations increased to approximately 1.8 mg/L (21). Donkeys have also been studied as a model for equines in response to gentamicin-induced acute kidney injury, with SAA levels among the parameters assessed. The results showed that mean SAA concentrations (along with other inflammatory parameters) were significantly higher at 7 and 14 days following gentamicin administration. However, the authors did not report the exact SAA concentration values (26). Previous work investigating the influences of acute bleeding on various blood parameters in donkeys. SAA concentrations did not change significantly from 30 min (287 ng/mL) to 240 min (213 ng/mL) following the induced bleeding, compared to the baseline value (318 ng/mL) (24). Nevertheless, the study was conducted on a relatively small group of eight animals. Other studies focusing on physiologic stages have reported markedly higher SAA values in Ragusano peripartum jennies and neonatal donkey foals (25.95 and 37.44 μg/mL, respectively) (22). These results are consistent with the concept that peripartum tissue remodeling and neonatal adaptation can activate inflammatory pathways even in clinically healthy animals. Field studies in recently captured feral donkeys measured with a stall-side method have shown mean SAA values in the mg/L range (10.8 mg/L at the initial sampling point and 17.4 mg/L at the second sampling) (14). Notably, samples were taken shortly after capture and relocation. Thus, these SAA concentrations may be compatible with a heightened inflammatory or stress response in that context (23).

Comparison with previously published donkey data highlights an important practical issue: SAA concentrations may differ substantially depending on the analytical method, calibration, sample matrix, and study population (14, 21, 22). Therefore, direct numerical comparisons between studies are difficult, and results should be interpreted using assay- and laboratory-specific reference intervals rather than a universal donkey threshold. In addition to analytical variability, differences in breed, environment, husbandry, workload, and exposure to subclinical inflammatory stimuli may also affect baseline SAA concentrations (10, 21, 28). These factors emphasize that reference intervals should be interpreted in relation to the population and conditions in which they were established.

The lower end of the distribution also merits comment. Two donkeys in the present dataset had SAA concentrations of 0.00 ng/mL, which supports retaining very low or undetectable values as biologically plausible in clinically normal animals. However, in formal RI derivation, the lower reference limit should be determined statistically from the reference distribution rather than fixed *a priori* at 0. Accordingly, the lower nonparametric reference limit in the primary analysis was 2.91 ng/mL, even though the observed minimum was 0.00 ng/mL. Very low or even undetectable SAA concentrations have also been reported in clinically healthy horses (15, 23, 29). However, direct numeric comparison between horse and donkey studies should be made cautiously because assays, matrices, calibration procedures, and reported units differ markedly across reports. This corresponds with our results, indicating that SAA concentrations in healthy donkeys may likewise be low. However, studies have demonstrated differences between donkeys and horses in the magnitude and kinetics of the SAA to inflammatory stimuli, reinforcing the need for species-specific RIs (10, 21, 30). From a clinical perspective, however, the presence of these higher values underscores that SAA should be interpreted in context and that repeat testing and concurrent clinical assessment remain important, particularly when results fall near or above the upper reference limit (31).

Several limitations should be considered. First, animals were sampled from a pasture-based management system during a single season; reference values may differ under alternative feeding systems, workloads, climates, or seasons. Second, although clinical examination,routine hematological evaluation, and biochemical tests were used to define health status, occult inflammatory conditions cannot be fully excluded without more extensive diagnostics. Third, the current study provides an RI for a specific ELISA platform; transferability to other assays is not guaranteed, and method comparison studies would be valuable. Future work should (i) evaluate biological variation and the potential need for partitioning by sex, age, breed, and physiologic status, (ii) assess diagnostic performance of SAA for common donkey diseases (e.g., colic, pneumonia, endotoxemia, postoperative inflammation), and (iii) establish clinically meaningful decision limits and/or delta-check approaches based on outcome-linked datasets.

In conclusion, this study provides an ASVCP-compliant, assay-specific, nonparametric RI for serum SAA in apparently healthy donkeys measured with a donkey-specific ELISA. The primary RI derived from the full reference cohort was 2.91–42.85 ng/mL. Because the upper limit was sensitive to high-end observations, this interval should be interpreted in the context of the analytical platform, population, and clinical findings, and should ideally be refined in future external validation studies.

## Data Availability

The original contributions presented in the study are included in the article/Supplementary material, further inquiries can be directed to the corresponding author.
