# Supplementary material for: Reference interval for serum amyloid a in apparently healthy donkeys measured with a donkey-specific ELISA
Source: Front Vet Sci. 2026 Jul 13;13:1884314. doi: 10.3389/fvets.2026.1884314 (PMC13402198; doi:10.3389/fvets.2026.1884314)
Supplement: Supplementary file 1 [file Table_1.DOCX]

Supplementary Material

**Supplementary Table S1. Intra-assay precision of ELISA validation samples**

| **Level** | **n** | **Mean ± SD** | **CV (%)** |
| --- | --- | --- | --- |
| Low | 4 | 3.63 ± 0.18 | 4,85 |
| Medium | 4 | 8.63 ± 1.06 | 12,30 |
| High | 4 | 11.23 ± 0.53 | 4,68 |
